# Palmitate and group B *Streptococcus* synergistically and differentially induce IL-1β from human gestational membranes

**DOI:** 10.3389/fimmu.2024.1409378

**Published:** 2024-05-23

**Authors:** Jennifer A. Gaddy, Rebecca E. Moore, Jonathan S. Lochner, Lisa M. Rogers, Kristen N. Noble, Ayush Giri, David M. Aronoff, David Cliffel, Alison J. Eastman

**Affiliations:** ^1^ Department of Medicine, Vanderbilt University Medical Center, Nashville, TN, United States; ^2^ Tennessee Valley Healthcare Systems, Department of Veterans Affairs, Nashville, TN, United States; ^3^ Publications Division, American Chemical Society, Washington, DC, United States; ^4^ Department of Microbiology, Immunology and Tropical Medicine, George Washington University, Washington, DC, United States; ^5^ Department of Pediatrics, Vanderbilt University Medical Center, Nashville, TN, United States; ^6^ Department Internal Medicine, Indiana University School of Medicine, Indianapolis, IN, United States; ^7^ Department of Obstetrics and Gynecology, Vanderbilt University Medical Center, Nashville, TN, United States; ^8^ Department of Chemistry, Vanderbilt University, Nashville, TN, United States

**Keywords:** pregnancy, gestational membranes, group B *Streptococcus*, obesity, palmitate, preterm prelabor rupture of membranes, preterm birth, inflammation

## Abstract

**Introduction:**

Rupture of the gestational membranes often precedes major pregnancy complications, including preterm labor and preterm birth. One major cause of inflammation in the gestational membranes, chorioamnionitis (CAM) is often a result of bacterial infection. The commensal bacterium *Streptococcus agalactiae*, or Group B *Streptococcus* (GBS) is a leading infectious cause of CAM. Obesity is on the rise worldwide and roughly 1 in 4 pregnancy complications is related to obesity, and individuals with obesity are also more likely to be colonized by GBS. The gestational membranes are comprised of several distinct cell layers which are, from outermost to innermost: maternally-derived decidual stromal cells (DSCs), fetal cytotrophoblasts (CTBs), fetal mesenchymal cells, and fetal amnion epithelial cells (AECs). In addition, the gestational membranes have several immune cell populations; macrophages are the most common phagocyte. Here we characterize the effects of palmitate, the most common long-chain saturated fatty acid, on the inflammatory response of each layer of the gestational membranes when infected with GBS, using human cell lines and primary human tissue.

**Results:**

Palmitate itself slightly but significantly augments GBS proliferation. Palmitate and GBS co-stimulation synergized to induce many inflammatory proteins and cytokines, particularly IL-1β and matrix metalloproteinase 9 from DSCs, CTBs, and macrophages, but not from AECs. Many of these findings are recapitulated when treating cells with palmitate and a TLR2 or TLR4 agonist, suggesting broad applicability of palmitate-pathogen synergy. Co-culture of macrophages with DSCs or CTBs, upon co-stimulation with GBS and palmitate, resulted in increased inflammatory responses, contrary to previous work in the absence of palmitate. In whole gestational membrane biopsies, the amnion layer appeared to dampen immune responses from the DSC and CTB layers (the choriodecidua) to GBS and palmitate co-stimulation. Addition of the monounsaturated fatty acid oleate, the most abundant monounsaturated fatty acid in circulation, dampened the proinflammatory effect of palmitate.

**Discussion:**

These studies reveal a complex interplay between the immunological response of the distinct layers of the gestational membrane to GBS infection and that such responses can be altered by exposure to long-chain saturated fatty acids. These data provide insight into how metabolic syndromes such as obesity might contribute to an increased risk for GBS disease during pregnancy.

## Introduction

The onset of labor and/or childbirth prior to term is often preceded by the rupture of extraplacental, gestational (“fetal”) membranes (1–3). Preterm premature rupture of membranes (PPROM) is commonly associated with histological inflammation, a condition termed chorioamnionitis (CAM). Acute, neutrophilic CAM can be caused by infectious or non-infectious stimuli, but a significant infectious cause of CAM is the bacterium Group B *Streptococcus* (GBS) (4–9). Further consequences of GBS-associated CAM include maternal and fetal sepsis and stillbirth (10–16).

The gestational membranes differentiate along with the trophectoderm during early human pregnancy. Both these tissues contain multiple cell types derived from both the fetus and the mother. The placenta is largely made up of cytotrophoblasts (CTBs), a fetal cell type which differentiates into several distinct forms. The gestational membranes are comprised of an outermost layer, the decidua, dominated by maternal decidual stromal cells (DSCs); a central chorion layer of fetal CTBs abutting the decidua; an inner amnion comprised of fetal mesenchymal cells, and a single layer of fetal amnion epithelial cells (AECs) lining the innermost (fetal-facing) aspect of the membranes. The amnion has ample extracellular matrix and basement membrane for structural stability. Macrophages are the principle resident phagocyte within uninflamed gestational membranes and there are cells of the adaptive immune system there as well (17). The precise cause of membrane rupture during CAM remains to be determined, but it likely involves matrix metalloproteinases (MMPs) produced by structural and immune cells of the membranes, and possibly the bacteria themselves (18–20). Additionally, several pro-inflammatory cytokines such as IL-6, IL-1β, IL-8, and TNF-α have been associated with CAM and preterm birth (21, 22).

The interplay of saturated fatty acids, pregnancy, obesity, and inflammation is complex. Saturated fatty acids are associated with inflammation [reviewed in (23)]. In normal weight individuals consuming a western diet, the monounsaturated fatty acid oleate and the LCSFA palmitate comprise (on average) 31% and 27% of fatty acids in the blood stream, respectively. The composition of these fatty acids are changed during obesity, with palmitate being the most abundant fatty acid in circulation at 33%, representing approximately a 20% increase than in individuals of normal-weight (24, 25). Thus, palmitate and oleate are often used to model the fatty acid component of obesity *in vitro*. Furthermore, palmitate specifically has been associated with inflammation, adverse pregnancy outcomes, and in several other pregnancy-associated conditions, including gestational diabetes mellitus and preeclampsia (26–30). Pregnancy progressively increases the circulating concentrations of fatty acids to increase energy availability, among other effects (31). While we would expect the serum levels of palmitate to increase during pregnancies complicated with obesity similarly to non-pregnant conditions, the concentration of serum palmitate in obese and normal weight pregnancies is also impacted by fetal sex (32). For instance, the fatty acid profiles of maternal plasma in obese pregnant persons differs from normal weight controls in distinct ways depending on fetal sex: obese pregnancies with female fetuses have higher maternal plasma oleate levels relative to controls, while obese pregnancies with male fetuses have lower levels of oleate than controls (32). There are also differences in unsaturated fatty acids between male and female placentas (33).

An estimated 1 in 4 pregnancy complications can be tied to obesity (34, 35), and this number is expected to rise as obesity rates increase. During non-pregnant settings, individuals with obesity are at an increased risk of rectovaginal colonization with GBS (36–38) and thus at greater risk of GBS-related pregnancy complications including CAM, preterm prelabor rupture of membranes, and fetal sepsis (39, 40). The precise cause of greater GBS colonization in obese pregnant people is unknown; however, the contribution of LCSFAs to inflammation has been well established (41).

In this study, we sought to determine the impact of the LCSFA palmitate on GBS-induced inflammation in different compartments of the gestational membranes. We were particularly interested in the potential for synergy between GBS and palmitate in the induction of proinflammatory cytokines, chemokines, and MMP production throughout the gestational membranes, since these peptides play significant roles in regulating the cellular inflammatory response to infection and the integrity of the extracellular matrix. We found that the DSC and CTB cell types displayed synergy in IL-1β secretion when treated with GBS and palmitate together. In contrast to previous work in the absence of palmitate that showed suppression of macrophage activation by both DSCs and CTBs (42), neither cell type inhibited activation of primary placental macrophages or macrophages of the cell line THP-1 in response to infection when palmitate was present. The AEC layer was largely nonresponsive to GBS and palmitate co-incubation and may actively inhibit the inflammation in the choriodecidua when the membranes are intact. The differences of each structural cell type of the gestational membrane in response to GBS and palmitate suggest complex regulation and that paracrine signaling between layers of the gestational membrane will be necessary.

## Materials and methods

### Cell lines and reagents

These studies used a telomerase-immortalized human endometrial stromal cell (THESC) uterine stromal cell line (obtained from ATCC, Manassas, VA, USA) differentiated into decidual stromal cells (DSC), as described below, the Jeg3 cytotrophoblast cell line (CTB, a kind gift from Dr. Carolyn Coyne), and the Tohoku Hospital Pediatrics-1 (THP-1) human monocytic leukemia-derived cell line (ATCC). DSCs were cultured in DMEM/F12 without phenol red (Invitrogen, Carlsbad, CA, USA) supplemented with 10% charcoal-stripped fetal bovine serum (csFBS, HyClone, Logan, UT, USA) and 1% antibiotic antimycotic solution (A/A) (Gibco, Waltham, Massachusetts, USA). We induced decidualization in the THESC line over 7 days with 0.5 mM 8-Br-cAMP,10 nM estradiol, and 1μM medroxyprogesterone acetate (Sigma-Aldrich, St. Louis, MS, USA) administered every 2 days to generate DSCs (43, 44). Induction of prolactin and IGFBP1 secretion, indicative of decidualization, was confirmed by ELISA (Alpha diagnostic international, San Antonio, TX, USA). CTBs were cultured in MEM without phenol red (Invitrogen) supplemented with 10% csFBS and 1% A/A. THP-1 cells were cultured in RPMI 1640 (Invitrogen) with 10% csFBS and 1% A/A. THP-1 cells were differentiated to adherent macrophage-like cells using phorbol myristate acetate (PMA, 5 ng/mL; Sigma, St. Louis, MO, USA) overnight, washing off nonadherent cells, and removing macrophages by incubating adherent cells using Cell Dissociation Buffer Enzyme-Free PBS-based (Gibco) for 5 minutes at 37°C and subsequent cell scraping.

Palmitate and oleate were obtained from Nu-Chek Prep (Elysian, MN, USA), dissolved in ethanol as in (45) and used at a final concentration of 0.4 mM without an added carrier. This concentration was selected based on circulating levels of palmitate in obese and/or diabetic individuals (39, 40) and to align with the palmitate doses employed in previous *in vitro* palmitate studies from a variety of groups (45–47). TLR4 agonist lipopolysaccharide (LPS, ultrapure from *E. coli* K12 strain) and TLR2 agonist PAM3CSK4 (PAM) were obtained from *In vivo*Gen (San Diego, CA, USA) and used at a final concentration of 1 μg/mL. The clinical GBS isolate GB00112 (GB112) was obtained from Dr. Shannon Manning (Michigan State University, East Lansing, MI, USA) (48). GBS was cultured in Todd-Hewitt broth (THB) overnight at 37°C, then pelleted and resuspended in PBS. GBS was used at a multiplicity of infection (MOI) of 10 for DSC, CTB, and AEC cells, and at an MOI of 100 for THP-1 cells. For experiments using whole or separated (amnion physically separated from choriodecidua) human gestational membrane punches, 1 x 10^7^ CFU of GBS was added to wells and thoroughly mixed.

### Human primary cell isolations

#### Human placental CTB isolation

These studies were conducted in accordance with the Vanderbilt University Institutional Review Board (IRB#181998) and Declaration of Helsinki. Pregnant people (aged 21-40 years) meeting our recruitment criteria receiving elective (scheduled) non-laboring but full-term Cesarean sections at Vanderbilt University Medical Center were approached and consented for donation of their placenta and gestational membranes. Exclusion criteria were: clinical evidence of CAM, immunocompromised conditions, collagen vascular disease, multi-fetal pregnancy, patients younger than 21 years of age or older than 40, evidence of bacterial vaginosis, cervical cerclage, third trimester bleeding, preeclampsia, diabetes (gestational or preexisting), SARS-CoV2 infection, or major medical conditions (chronic renal disease, sarcoidosis, hepatitis, HIV, etc). Demographics and known variables of patients are included in **Supplementary Table 1**.

#### Human placental macrophage isolation

Human placental macrophages from the villous core were isolated as previously described (49). Briefly, placental cotyledon tissue, separated from the maternal decidua basalis, was vigorously washed with PBS to remove circulating blood. The tissue was minced into small pieces and weighed to determine final grams collected. Tissue fragments were placed into 250 mL sterile bottles with sterile digestion solution containing 150 μg/mL deoxyribonuclease, 1 mg/mL collagenase, and 1 mg/mL hyaluronidase at 10 mL per gram of tissue. Placenta was digested for 1 h at 37°C, shaking at 180 RPM. Digested tissue was filtered through a 280 μm metal sieve, followed by 180 and 80 μm nylon screens. Cells were centrifuged at 1500 RPM and resuspended in 25% Percoll diluted in cold RPMI media containing 10% FBS and 1% A/A (referred hereafter as RPMI +/+) and overlaid onto 50% Percoll, plus 2 mL of PBS on top of the density gradient. CD14+ macrophages were isolated by positive selection after red blood cell (RBC) lysis (solution from Invitrogen) using the magnetic MACS^®^ large cell separation column system according to the manufacturer’s instructions. Isolated CD14+ placental macrophages were plated and rested overnight in RPMI+/+ at 37 °C with 5% CO2 before experimentation. Purity of placental macrophage preparations was determined to be 95.89 +/- 0.6986% based on CD68 positivity.

#### Human gestational membrane amnion epithelial cell isolation

Gestational membrane was physically separated into amnion and choriodecidua by peeling the layers from each other. Choriodecidua was discarded and amnion was cut into approximately 2-inch squares and added to a beaker containing 100mL warmed 0.25% Trypsin. Amnion was incubated in a shaking incubator at 200RPM at 37°C for 60 minutes. Amnion-trypsin solution was strained through a metal screen, flow-through containing AECs saved, amnion squares returned to beaker, and another 100 mL 0.25% trypsin was added. The beaker with amnion and trypsin was returned to shaking incubator for 30 minutes. Flow-through containing AECs was strained through 70 μm strainer and divided into 50-mL conical tubes resuspended in complete media. After 30 minute incubation, amnion-trypsin solution was filtered, newborn calf serum added to neutralize trypsin, flow-through was further strained through 70 μm screens, and spun to pellet AECs. Pellets were pooled, washed in PBS, spun down again to pellet, and subjected to red blood cell lysis as per manufacturer’s protocol. Cells were pelleted after RBC lysis step, resuspended in complete media, counted, and plated in 24-well dishes at a concentration of 1 x 10^6^/mL, 1 mL per well. Wells were washed on subsequent days and cells monitored until they formed a connected, confluent epithelial cell monolayer.

#### Human gestational membrane punch biopsy preparation

Human gestational membranes were washed in warm, sterile PBS to remove blood and clots. Membranes were spread over a sterile silicone pad and 12 mm punch biopsies were obtained. For experiments using separated amnion and choriodecidua, amnion was physically pulled from choriodecidua, then amnion and choriodecidual membrane layers were spread individually over the silicone pad and 12 mm biopsy punches made. Tissue punches were added to wells of 24-well plates with 1 mL complete DMEM/F12 media and rested overnight before infection.

### Fatty acid treatment and infection

Media was aspirated from wells, cells or tissue rinsed with sterile PBS, and then palmitate, oleate, vehicle control (ethanol), and/or GBS was added to selected wells at the same time in antibiotic-free media (RPMI 1640 or DMEM/F12, depending on cell type) with 10% csFBS. Cultures sat in antibiotic-free media with or without GBS and fatty acids for 1 hour. Antibiotic/antimycotic was added to wells to a concentration of 1x (from 100X stock), and then cultures were incubated for an additional 24 hours. Media was then harvested for ELISA.

### Enzyme-linked immunosorbent assay

ELISA analysis was performed on supernatants from cultures as per manufacturer’s protocol for each analyte (IL-1β, IL-8, TNFα, CCL2, CCL5, G-CSF, GM-CSF, and MMP9) (R&D systems, Minneapolis, MN, USA).

### Statistical analysis

All experiments were performed in multiple, independent biological replicates as noted in each figure. Data points from independent experiments are presented along with means and analyzed with GraphPad Prism 6.0 software (GraphPad Software, San Diego, CA). Data were analyzed by 2-way ANOVA with multiple comparisons. Differences were considered statistically significant for *p < 0.05*.

## Results

### Palmitate-supplemented media promotes GBS proliferation

**Figure 1:**
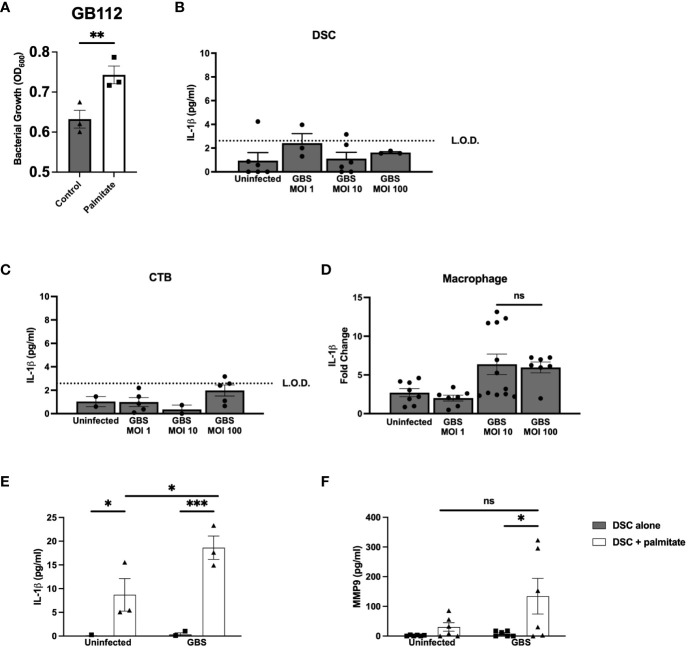
Long chain saturated fatty acid palmitate induces Group B *Streptococcus* growth, synergizes with GBS infection to induce IL-1β and MMP9 from human decidual stromal cells. **(A)** OD_600_ reading of GB112 grown for 24 hours in THB broth with and without added palmitate. IL-1β secretion measured by ELISA from the uterine stromal cell line THESC decidualized over 7 days with estrogen, progesterone, and cAMP (referred to as DSCs) **(B)**, the CTB cell line HTR-8 **(C)**, and the macrophage cell line THP.1 **(D)** infected with GBS for 24 hours at MOIs of 1, 10, and 100. **(E)** IL-1β and **(F)** MMP9 secretion measured by ELISA from uninfected or GBS-infected DSCs with or without added palmitate for 24 hours. For all experiments in **Figure 1**, N = 3 or more separate experiments with averaged independent replicates. *p < 0.05, **p < 0.01, ***p < 0.001 by Student’s T-test **(A–D)**, or 2-way ANOVA **(E, F)**. ns, not significant; L.O.D., limit of detection.

Prior to beginning experiments, we assessed whether palmitate affected GBS growth. After 24 hours, THB media supplemented with palmitate resulted in greater proliferation of GBS (**Figure 1A**) as estimated by spectrophotometry (OD_600_). To control for the potential GBS dosing differences between palmitate and non-palmitate groups, we assessed cytokine production at multiplicity of infections (MOIs) of 1, 10, and 100. Our standard MOI is 10, thus the addition of palmitate has the potential to increase this MOI. When we assayed supernatants of DSCs, CTBs, and THP-1 macrophages infected with different MOIs of GBS, we found no differences between the MOIs of 10 and 100 (**Figures 1B–D**), and thus have reason to believe that differences in cytokine production in the presence or absence of palmitate throughout the manuscript resulted from the presence of palmitate and not to changes in GBS MOI.

### Palmitate and GBS co-stimulation induces greater cytokine response in DSCs, CTBs, and macrophages than either treatment individually

To determine the effect of palmitate and GBS dual stimulation on the choriodecidua, we induced decidualization in DSCs as described in methods. DSC monocultures were then infected with GBS, treated with palmitate, neither, or both. In our initial cytokine screen, we found that IL-1β (**Figure 1E**) and CCL2 (**Supplementary Figure S1A**) were both induced by GBS infection and by palmitate treatment, and the combination of palmitate and GBS synergized, producing more than the simple summation of the IL-1β produced by each stimulus alone. MMP9 (**Figure 1F**), CCL5, and GM-CSF (**Supplementary Figures S1B, C**) were all induced by palmitate and GBS combined, but not by either stimulus alone, also consistent with a synergistic interaction. G-CSF was induced by palmitate both during GBS infection and when uninfected (**Supplementary Figure S1D**). Neither palmitate nor GBS significantly induced IL-8 or TNFα secretion, although there was a trend for increased production in the presence of palmitate in both cytokines (**Supplementary Figures S1E, F**).

When treated with PAM or LPS to assess the applicability of these findings to other pathogens, we observed similar, if not more dramatic, results in DSCs (**Supplementary Figure S2**). Palmitate synergized with both PAM and LPS induced greater IL-1β, IL-8, CCL5, G-CSF, and CCL2 secretion, while MMP9, GM-CSF, and TNFα levels did not change significantly between treatments (there were non-significant trends towards increases) (**Supplementary Figure S2**).

**Figure 2:**
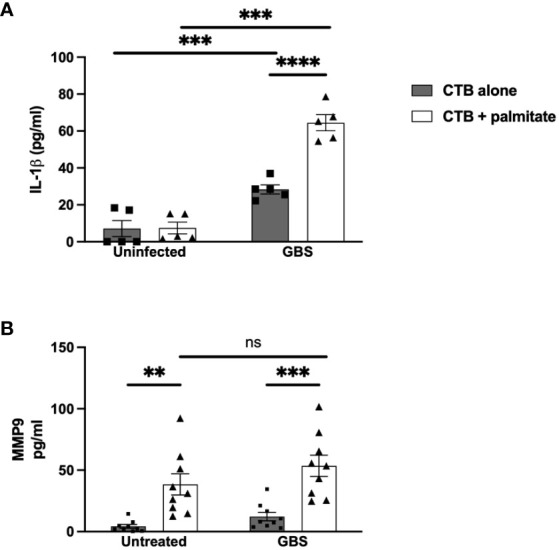
LCFA palmitate induces MMP9 secretion and synergizes with GBS to induce IL-1β secretion from human cytotrophoblasts. **(A)** IL-1β and **(B)** MMP9 secretion measured by ELISA from cytotrophoblast cell line HTR8 uninfected or GBS-infected with or without added palmitate for 24 hours. N = 3 or more separate experiments with averaged independent replicates. **p < 0.01, ***p < 0.001, ****p < 0.0001 by 2-way ANOVA. ns, not significant.

Moving inwards to cell types of the chorion, we used the Jeg3 cell line of CTBs as a model of chorionic trophoblasts, to assess the impact of palmitate and GBS dual stimulation on the cytokine response in CTBs alone. Narrowing the field of proteins measured to focus on MMP9 and IL-1β, we found that palmitate stimulation alone did not induce IL-1β secretion as in the DSCs (**Figure 2A**), but GBS infection did induce IL-1β production both with and without palmitate, and palmitate stimulation further increased GBS-induced IL-1β production in CTBs (**Figure 2A**). Similar to the DSCs, palmitate induced MMP9 production in the presence or absence of infection (**Figure 2B**). Stimulation of CTBs with palmitate and TLR ligands PAM or LPS synergistically induced greater TNFα, MMP9, and GM-CSF production (**Supplementary Figure S3**).

**Figure 3:**
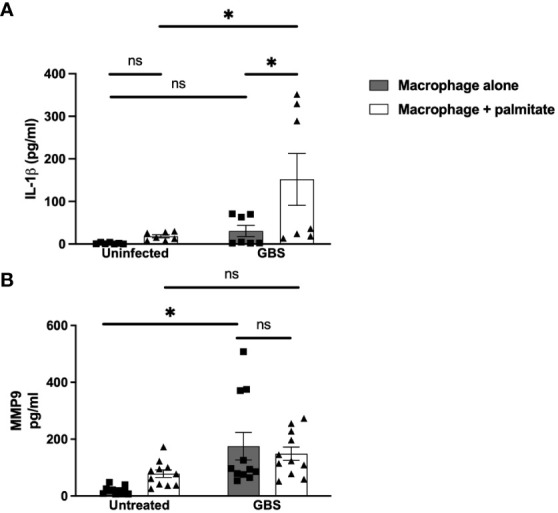
LCFA palmitate synergizes with GBS to induce IL-1β secretion from macrophages but not MMP9. **(A)** IL-1β and **(B)** MMP9 secretion measured by ELISA from PMA-stimulated human monocyte cell line THP-1 uninfected or GBS-infected with or without added palmitate for 24 hours. N = 5 or more separate experiments with averaged independent replicates. *p < 0.05 by 2-way ANOVA. ns, not significant.

We modeled the macrophage response to GBS and palmitate stimulation using the macrophage-like cell line THP-1 differentiated with PMA. Similar to both the DSCs and CTBs, GBS and palmitate stimulation increased IL-1β cytokine production significantly more than either stimulation alone (**Figure 3A**). MMP9 was induced by GBS stimulation with no significant increases in the presence of palmitate (**Figure 3B**). Macrophage responses to the combination of PAM or LPS varied depending on the analyte: while GM-CSF was induced synergistically when co-stimulated with PAM or LPS and palmitate, MMP9 and TNFα varied: PAM treatment resulted in additive effects for TNFα while LPS did not, and LPS resulted in additive effects for MMP9 while PAM did not (**Supplementary Figure S4**).

### Palmitate and GBS co-stimulation modulates cell-cell interactions

**Figure 4:**
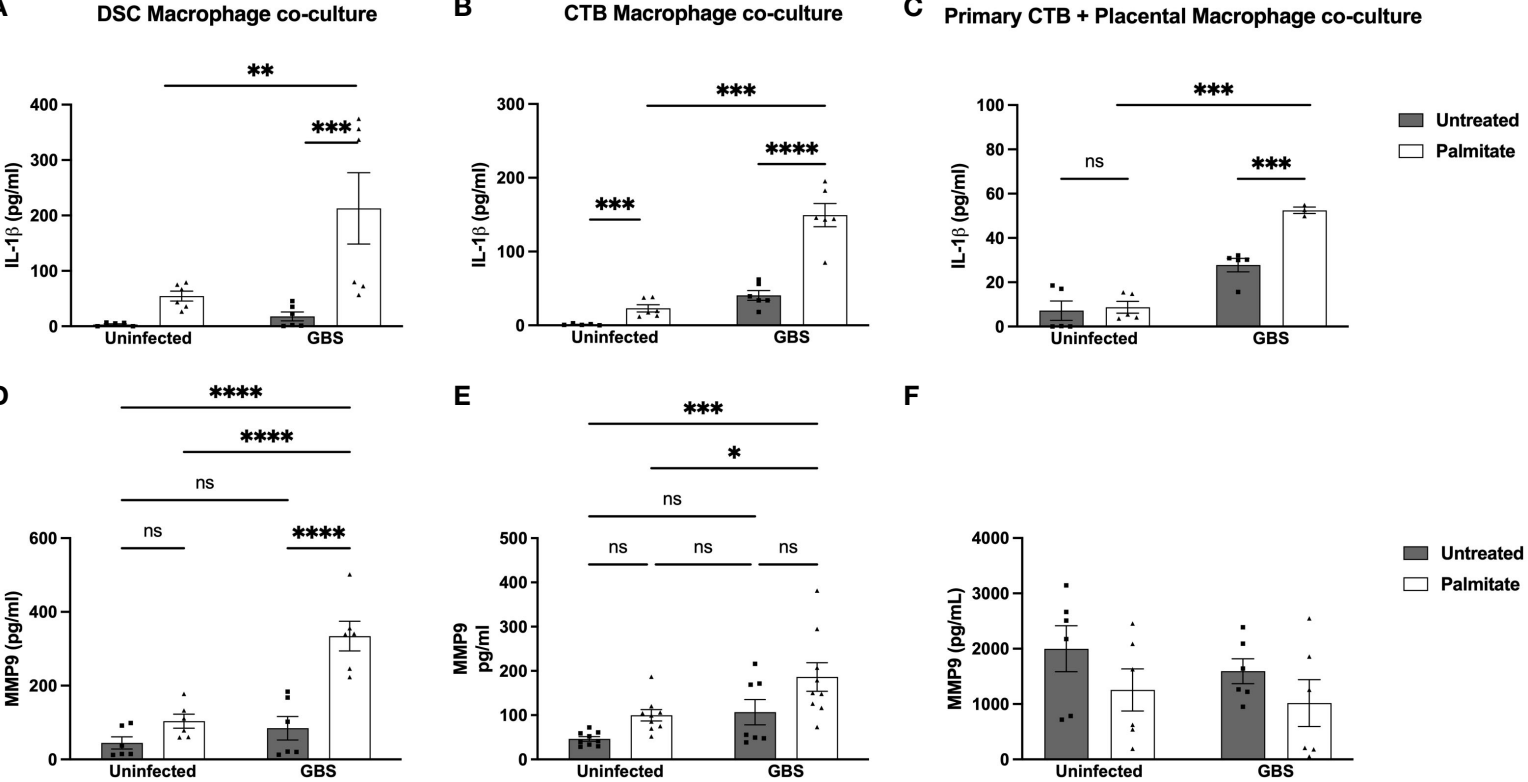
Palmitate and GBS synergize to induce IL-1β secretion from DSC-Mφ and CTB-Mφ co-cultures, while MMP9 secretion is more complicated. **(A–C)** IL-1β and **(D–F)** MMP secreted protein by ELISA in co-cultures of cell line and primary structural and immune cells. **(A, D)** ELISA data of co-cultures of DSCs with THP-1 Mφ over 24hrs of palmitate treatment and GBS infection. **(B, E)** Co-cultures of HTR8 CTBs and THP-1 Mφs over 24hrs of palmitate treatment and GBS infection. **(C, F)** primary placental CTBs co-cultured with primary placental Mφ over 24hrs of palmitate treatment and GBS infection. N = minimum of 4 separate experiments with averaged independent replicates. *p < 0.05, **p < 0.01, ***p < 0.001, ****p < 0.0001 by 2-way ANOVA. ns, not significant.

DSCs and CTBs can suppress macrophage cytokine production (42, 50). To assess whether this happens in the context of IL-1β or MMP9 in the presence of GBS and/or palmitate, we combined immune and structural cells (decidualized THESC cells (DSCs) or CTBs (Jeg3 cell line)) with THP-1 macrophages at a ratio of 10 structural cells to 1 macrophage (10:1). We found that IL-1β was still significantly induced by GBS and palmitate during co-culture (**Figures 4A, B**), and not suppressed. However, neither co-culture system appeared to augment IL-1β production over what macrophages alone produced (**Figure 3**). When using primary CTBs with primary placental macrophages instead of Jeg3 CTBs and THP-1 macrophages, we found that palmitate and GBS also induced IL-1β synergistically, but that the combination of CTBs with primary placental macrophages was not synergistic (**Figure 4C**). In the co-culture system, MMP9 was induced significantly in DSC-macrophage co-culture only during GBS and palmitate treatment together (**Figure 4D**). GBS and palmitate together increased MMP9 expression over baseline in CTB-macrophage co-culture (**Figure 4E**). In primary placental CTB and macrophage co-cultures, MMP9 was not induced by any treatment and in fact trends towards suppression with GBS and palmitate treatment (**Figure 4F**).

### Whole and separated human gestational membrane punches exhibit a range of responses to GBS and palmitate co-stimulation

**Figure 5:**
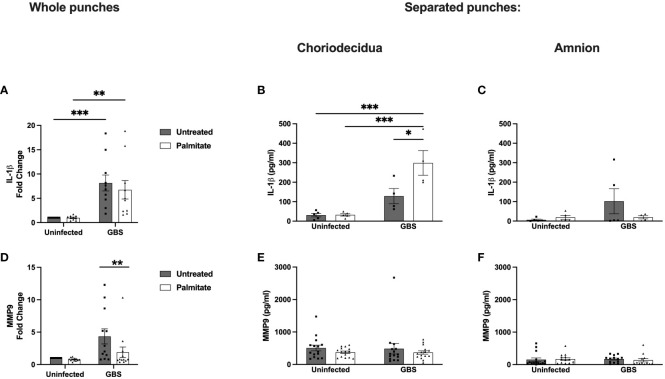
Whole and separated human extraplacental membrane punches have differential IL-1β and MMP9 responses to palmitate and GBS. **(A–C)** IL-1β and **(D–F)** MMP9 secretion from whole and separated human extraplacental membrane punches after 24 hours of culture with GBS and palmitate. **(A, D)** Whole membrane punches show increase in IL-1β and MMP9 during GBS infection that is not tied to palmitate treatment. **(B)** GBS and palmitate synergize in the choriodecidua to induce IL-1β, while there is no induction of MMP9 secretion **(E)** from any treatment. **(C, F)** Amnion punches do not induce IL-1β or MMP9 significantly under any treatments. N = minimum of 3 separate experiments with averaged independent replicates. *p < 0.05, **p < 0.01, ***p < 0.001 by 2-way ANOVA.

We next assessed the IL-1β and MMP9 responses to GBS and palmitate in whole human gestational membrane biopsy specimens obtained from term, nonlaboring c-sections. Interestingly, we found that GBS induced IL-1β expression, but palmitate addition to GBS did not synergistically induce IL-1β (**Figure 5A**). To tease apart this finding, we matched the data from each untreated membrane punch to its own corresponding palmitate-stimulated membrane punch (e.g. punched from the same patient’s membranes) in both infected and uninfected conditions. During uninfected conditions, there was very little induction of IL-1β by palmitate, and palmitate-stimulated IL-1β levels were on a spectrum ranging from suppression to induction (**Figure 5A**). During GBS stimulation, there was a clear divide in punches from the same membranes with and without palmitate: roughly equal numbers of punches had their IL-1β induced as had IL-1β suppressed by palmitate during GBS infection (**Supplementary Figure S5A**). Organizing the data by maternal body-mass index (BMI), fetal sex, age, or GBS colonization status was unable to resolve the discrepancy (data not shown).

To resolve the discrepancy in IL-1β production from whole membrane punch responses to GBS and palmitate compared to isolated cell lines and co-cultures, we next separated amnion from choriodecidua and repeated the experiment quantifying IL-1β from culture supernatants (**Figures 5B, C**; **Supplementary Figure S5C**). In the choriodecidua, GBS and palmitate synergized to induce IL-1β as seen in the cell lines. Conversely, the amnion did not show significant IL-1β induction under any condition and in fact, palmitate treatment trended non-significantly towards suppression of IL-1β (**Figure 5C**).

MMP9 production was induced 5-fold with GBS stimulation alone in whole membrane punches, which was significantly greater than the 2.5-fold increase with GBS and palmitate together (**Figure 5D**). The response of paired samples showed a consistent pattern of each patient’s MMP9 secreted from membrane punches decreasing with palmitate addition (**Supplementary Figure S5B**). We next assessed the contribution of the choriodecidua versus the amnion in this response and found that no conditions alone or together induced MMP9 from either the choriodecidua or the amnion (**Figures 5E, F**; **Supplementary Figure S5D**). This was distinct from the results obtained with cell lines (**Figures 1**, **2**).

### AECs are largely nonresponsive to individual or co-treatment with GBS or palmitate

**Figure 6:**
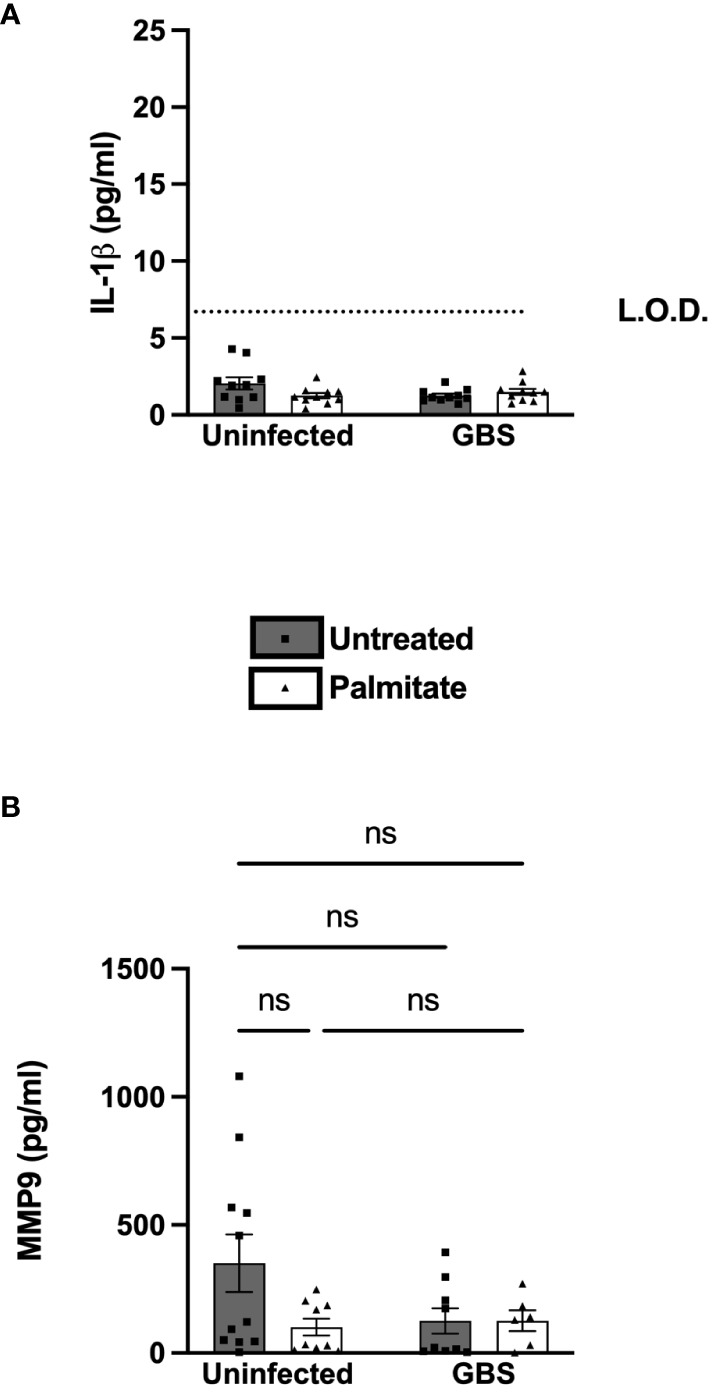
Primary human amnion epithelial cells (AECs) do not induce IL-1β or MMP9 under any treatments. **(A)** IL-1β and **(B)** MMP9 secretion measured by ELISA from uninfected or GBS-infected AECs with or without added palmitate for 24 hours. For all experiments, N = 4 or more separate experiments with averaged independent replicates. ns, not significant 2-way ANOVA. L.O.D., limit of detection.

We next determined the response of primary amnion epithelial cells (AECs) obtained from human placenta to GBS and/or palmitate. The AECs did not induce IL-1β over the limit of detection in response to GBS or palmitate alone or in combination (**Figure 6A**). However, there was a nonsignificant trend towards suppressed MMP9 in all treatment groups relative to controls, similar to the nonsignificant MMP9 trend seen in primary CTB-Mφ co-cultures (**Figure 6B**).

### Addition of the unsaturated fatty acid oleate blocks palmitate and GBS-associated IL-1β induction

**Figure 7:**
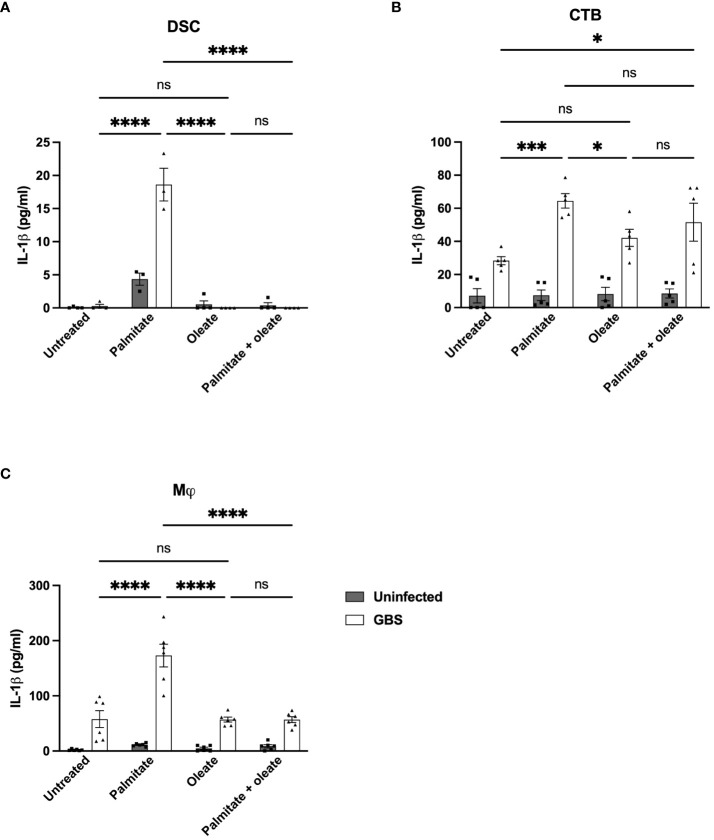
Oleate ameliorates the synergistic induction of IL-1β from palmitate and GBS dual stimulation in DSC, CTB, and Mj cell lines. IL-1β secretion measured by ELISA from **(A)** DSC, **(B)** CTB, and **(C)** THP-1 Mφ when infected with GBS and treated with palmitate and/or oleate. N = minimum of 4 separate experiments with averaged independent replicates. *p < 0.05, ***p < 0.001, ****p < 0.0001 by 2-way ANOVA. ns, not significant.

There are reports in the literature that unsaturated fatty acids can alleviate palmitate-induced changes to inflammation (51). We thus added the unsaturated fatty acid oleate to DSC, CTB, and THP-1 macrophage cultures with and without GBS infection and palmitate treatment and measured IL-1β secretion. We did not measure MMP9 with oleate due to the inconsistent induction in the presence of palmitate across the cell lines used. In DSCs, palmitate and GBS still synergistically induced IL-1β, oleate did not induce IL-1β, and the addition of oleate to palmitate was sufficient to significantly suppress palmitate-induced IL-1β secretion (**Figure 7A**). In CTBs, palmitate and GBS synergistically induced IL-1β, while the addition of oleate did not. The addition of oleate to palmitate during GBS infection was unclear: oleate and palmitate during GBS infection did not differ significantly from oleate and GBS stimulation or palmitate and GBS stimulation, while oleate/GBS and palmitate/GBS did differ significantly (**Figure 7B**). Finally, THP-1 macrophages stimulated with palmitate and GBS synergistically induced IL-1β, while oleate and GBS did not induce IL-1β, and, similarly to the DSCs, the addition of oleate to palmitate during GBS infection suppressed IL-1β production (**Figure 7C**).

## Discussion

In this manuscript, we have shown *in vitro* and ex vivo that distinct layers of the gestational membranes respond differently to the saturated fat palmitate in the presence or absence of the common vaginal commensal bacterium GBS. We demonstrate that palmitate enhances GBS proliferation (**Figure 1**), which may partially explain the increased complications due to GBS in people with obesity during pregnancy, due simply to the increased proliferation of GBS (52, 53). While independent layers in the choriodecidua respond to GBS and palmitate stimulation with synergy in cytokine and MMP induction, the amnion epithelium does not contribute to this phenotype and is largely unaffected by GBS and palmitate stimulation, both in isolated primary amnion epithelial cells and in gestational membrane punch biopsies, where the amnion has been separated from the choriodecidua. This was not surprising, as the broad dampening of inflammation by the amnion is well-established (54–56). Furthermore, we have shown that macrophages (either cell line or primary placental macrophages) co-cultured with structural cells of the choriodecidua in the presence of palmitate and GBS are not suppressed by the structural cells as can be seen in the absence of palmitate in previous work (42, 50). Different patterns of analyte induction in the presence or absence of GBS-palmitate synergy may be due to signaling and transcription factors controlling the particular analytes in a particular cell type; for instance, while IL-1β is reliably induced synergistically from GBS and palmitate in combination, MMP9 synergy is less reliable. IL-1β is controlled at many levels and processed from a pro-form to an active form, while MMP9 is largely controlled at the transcriptional level with the association of coactivators distinct from IL-1β (57). Synergy between palmitate and GBS stimulation likely depends entirely on the signaling pathways to, and regulation of, each particular analyte. Sensitization deriving from one stimulus then augmenting other stimuli has been shown during pregnancy before (58–62), although this has largely been shown for the interplay between viral stimulation or viral PAMPs and subsequent stimulation.

This synergistic production of cytokines and MMPs is not specific to live GBS, as shown by the ability of a TLR4 agonist (LPS) to induce synergy in cytokine expression when administered with palmitate (**Supplementary Figure S2**). Because we observed cytokine augmentation when palmitate was combined with live GBS [a gram-positive bacterium) as well as a purified TLR4 agonist (LPS, a gram-negative pathogen-associated molecular pattern (PAMP)], this suggests broad applicability of our findings to different bacterial species and strains, both gram positive and gram negative. Further, it suggests that palmitate-induced alterations to GBS proliferation and metabolism are not the primary cause of increased inflammation in the gestational membranes, since the synergistic inflammation occurs with isolated PAMPs as well. We observed a relative inability of TLR2 agonist PAM3CSK4 to induce cytokines or MMPs on its own or in combination with palmitate when applied to DSCs. This lack of stimulation was recapitulated in CTBs as well (**Supplementary Figure S3**), with the exception of GM-CSF, where PAM3CSK4 was able to induce GM-CSF alone and synergize with palmitate. However, PAM3CSK4 was able to induce several cytokines alone (IL-1β, CCL5, GM-CSF), but not MMP9, and synergize with palmitate in THP-1 macrophages similar to live GBS and purified LPS (**Supplementary Figure S4**). Additional experimentation needs to be carried out to address the discrepancy between TLR2 activation and palmitate synergy in structural cells versus immune cells (such as macrophages) to determine the mechanism(s) at play, such as specific pathway activation or suppression, or receptor expression. Additional approaches could include using Pam2Cys or lipoteichotic acid (LTA), which utilize a different heterodimer of TLR2; while PAM3CSK4 utilizes a TLR1-TLR2 heterodimer, LTA and Pam2Cys utilize a TLR2-TLR6 heterodimer. The discrepancy between PAM3CSK4 and GBS-induced activation of cytokine and MMP may have to do with the activation of the TLR1/2 versus TLR2/6 heterodimers (63).

There are many varied fatty acids in the bloodstream, and one limitation of this study is the use of palmitate in isolation, or in combination with a single unsaturated fatty acid (oleate), instead of looking at the effects of a cocktail of saturated and unsaturated fatty acids in varying proportions on the cell types found within the gestational membrane. We acknowledge this shortcoming as a necessary oversimplification for the purposes of *in vitro* experimentation. Palmitate was chosen as it is the most abundant saturated fatty acid in circulation during obesity and can be the dominant circulating fatty acid. Furthermore, it is the most common saturated fatty acid used for *in vitro* experiments of obesity in the literature, and as such there is a large body of knowledge surrounding its mechanisms, making it an ideal candidate to study our models presented here in a reductionist manner. Continuing this line of inquiry using distinct mixtures of fatty acids, and the use of our previously-published metabolic cocktail (glucose, insulin, palmitate) (64) will more accurately reflect the milieu seen *in vivo*. However, defining basic patterns of synergy between infectious stimuli and a fatty acid using a reductionist approach gives us a base from which to determine the relative contributions of different fatty acids and sugars to the inflammatory state of the gestational membranes. The mechanism behind palmitate-induced inflammation remains unclear. There is much discrepancy in published work as to whether palmitate can signal through TLR4 (65–71) or not (72). As such, the mechanism behind synergy of GBS and palmitate is also unclear, but it is likely that palmitate stimulates a pathway or receptor independent of TLR4 signaling because palmitate treatment augments the inflammatory response to TLR4 agonists in macrophages, CTBs, and LPS (**Supplementary Figures S2**-**S4**). This includes the induction of: IL-1β, IL-8, CCL2, CCL5, and G-CSF in DSCs; TNFα, MMP9, and GM-CSF in CTBs; and MMP9 and GM-CSF in macrophages.

The condition of “diabesity”, a combination of type-2 diabetes and obesity, may more accurately reflect the reality of which pregnant individuals experience a greater rate of pregnancy complications than BMI alone. The inability of BMI to assess body fat distribution (abdominal versus distributed) or muscle mass adds heterogeneity into the system. Additionally, metabolic syndrome, a classification that encompasses the risk of developing type-2 diabetes and cardiovascular disease, can be found in individuals with any BMI (73). Access to patient electronic medical records containing bloodwork results such as circulating hemoglobin A1C or lipids, or a waist-to-hip ratio, could more accurately stratify our human gestational membrane punch data to reveal patterns that maternal BMI does not.

We expected to see an increase in inflammatory mediators upon co-stimulation of whole human gestational membrane punches with palmitate and GBS. However, for the analytes we selected for these experiments, we were unable to find consistent patterns or statistical significance between treatments. We had 11 patients and 24 membrane punches for each patient allowing for biological and technical replicates for each patient. As shown in **Supplementary Table 1**, the BMI of most patients was between the overweight and morbidly obese categories by BMI. However, 1) there was not a direct relationship between BMI and magnitude of cytokine induction (data not shown), and 2) palmitate and GBS did synergize in IL-1β production from the membrane punches of roughly half the patients, while the other half of patients’ membrane punches showed no synergy or decreased production upon palmitate stimulation. We were similarly unable to show a direct correlation between BMI and these cytokine induction patterns. Sorting by fetal sex, maternal GBS status, ethnicity, or gestational age also did not uncover any correlation between magnitude or direction of cytokine induction. It is very possible that with a larger N value of patient samples, the impact of particular patient variables could be observed. Recent studies have found that male placentas from pregnancies of obese people had significantly increased levels of fetal placental macrophages, which could easily have an impact on inflammatory responses in the gestational membranes (74). The gestational membrane punch biopsies we used, even when separated, likely contained up to 13% immune cells (75). And fetal sex also has an impact on how many and what kind of circulating maternal fatty acids accumulate in the placenta (32, 33), which could add another confounder to data from human gestational membranes: the impact of increased saturated fatty acids (already pro-inflammatory) and unsaturated fatty acids (which may be anti-inflammatory) already present in the membrane punches.

Additionally, a better understanding of the metabolic conditions that drive the molecular underpinnings of infection and inflammation during pregnancy can inform immunological interventions to ameliorate the risk of adverse perinatal outcomes. IL-1β is a tantalizing target because this molecule has been implicated as an important biomarker associated with chorioamnionitis, PPROM, and preterm labor (21). IL-1β is a pro-inflammatory cytokine that perturbs maternal-fetal tolerance (76). Our work demonstrates that palmitate and GBS infection promote IL-1β production, underscoring the role both metabolic dysfunction and infection could play in pregnancy homeostasis. There is an association between absolute levels of IL-1β and pregnancy complications: as levels of IL-1β increase, the likelihood of preterm deliveries increases (77). Accordingly, there is likely biological relevance for the synergy of GBS and palmitate: it is not just presence or absence of IL-1β that can result in pregnancy complications, but the greater the IL-1β levels, the greater the likelihood of pregnancy complications, and thus the combination of palmitate and GBS inducing more IL-1β in than either one alone would predispose pregnant people with both stimuli to a greater likelihood of pregnancy complications.

## Data availability statement

The raw data supporting the conclusions of this article will be made available by the authors, without undue reservation.

## Ethics statement

The studies involving humans were approved by Vanderbilt University Institutional Review Board. The studies were conducted in accordance with the local legislation and institutional requirements. The participants provided their written informed consent to participate in this study.

## Author contributions

JG: Funding acquisition, Methodology, Resources, Supervision, Writing – original draft, Writing – review & editing. RM: Investigation, Writing – original draft, Writing – review & editing. JL: Investigation, Writing – review & editing. LR: Investigation, Writing – review & editing. KN: Investigation, Writing – review & editing. AG: Formal analysis, Methodology, Writing – review & editing. DA: Funding acquisition, Methodology, Resources, Supervision, Writing – review & editing. DC: Funding acquisition, Resources, Supervision, Writing – review & editing. AE: Conceptualization, Data curation, Formal analysis, Funding acquisition, Investigation, Methodology, Project administration, Resources, Visualization, Writing – original draft, Writing – review & editing.

## Supplementary material

Supplementary Figure 1 Cytokine induction by GBS, palmitate, or both in DSCs infected with GBS and/or treated with palmitate. ELISA analysis of secreted cytokines from DSCs. **(A)** CCL2, **(B)**, CCL5, **(C)** GM-CSF, **(D)** G-CSF, **(E)** IL-8, and **(F)** TNFα each followed different patterns of induction by 24 hour infection and/or treatment with GBS and/or palmitate. N = minimum of 3 separate experiments with averaged independent replicates. *p < 0.05, **p < 0.01, ****p < 0.0001 by 2-way ANOVA. ns, not significant.

Supplementary Figure 2 Secreted inflammatory protein induction by TLR2 and 4 agonists in conjunction with palmitate in DSCs. **(A)** IL-1β, **(B)** MMP9, **(C)** IL-8, **(D)** CCL2, **(E)** CCL5, **(F)** GM-CSF, **(G)** G-CSF, **(H)** TNFα each follow different patterns of induction by 24hr stimulation with TLR2 and 4 ligands PAM3CSK4 (PAM) and LPS, respectively. N = minimum of 3 separate experiments with averaged independent replicates. *p < 0.05, **p < 0.01, ****p < 0.0001 by 2-way ANOVA. ns, not significant.

Supplementary Figure 3 Secreted inflammatory protein induction by TLR2 (PAM) and 4 (LPS) agonists in conjunction with palmitate in CTBs. **(A)** TNFα, **(B)** MMP9, and **(C)** GM-CSF. N = minimum of 3 separate experiments with averaged independent replicates. *p < 0.05, **p < 0.01, ****p < 0.0001 by 2-way ANOVA. ns, not significant.

Supplementary Figure 4 Secreted inflammatory protein induction by TLR2 (PAM) and 4 (LPS) agonists in conjunction with palmitate in THP-1 macrophages. **(A)** TNFα, **(B)** MMP9, and **(C)** GM-CSF. N = minimum of 3 separate experiments with averaged independent replicates. *p < 0.05, **p < 0.01, ****p < 0.0001 by 2-way ANOVA. ns, not significant.

Supplementary Figure 5 Matched sample and side-by-side comparison of IL-1β and MMP9 secretion from membrane punches from human extraplacental membrane.**(A)** IL-1β and **(B)** MMP9 ELISAs of 24hr supernatants from individual patient’s membrane punches matched with line between treatments with GBS and palmitate. **(C)** IL-1β and **(D)** MMP9 ELISA of CHD and AMN for side-by-side comparison of protein secretion between adjacent extraplacental membrane layers. **(A, B)** N = 11 independent experiments with averaged independent replicates; **(C, D)** N = 5 independent experiments with averaged independent replicates. * p < 0.05, **p < 0.01, ***p < 0.001, ****p < 0.0001 by 2-way ANOVA. ns, not significant.
