# Supplementary figures and images for: Palmitate and group B Streptococcus synergistically and differentially induce IL-1β from human gestational membranes
Source: Front Immunol. 2024 May 23;15:1409378. doi: 10.3389/fimmu.2024.1409378 (PMC11158625; doi:10.3389/fimmu.2024.1409378)

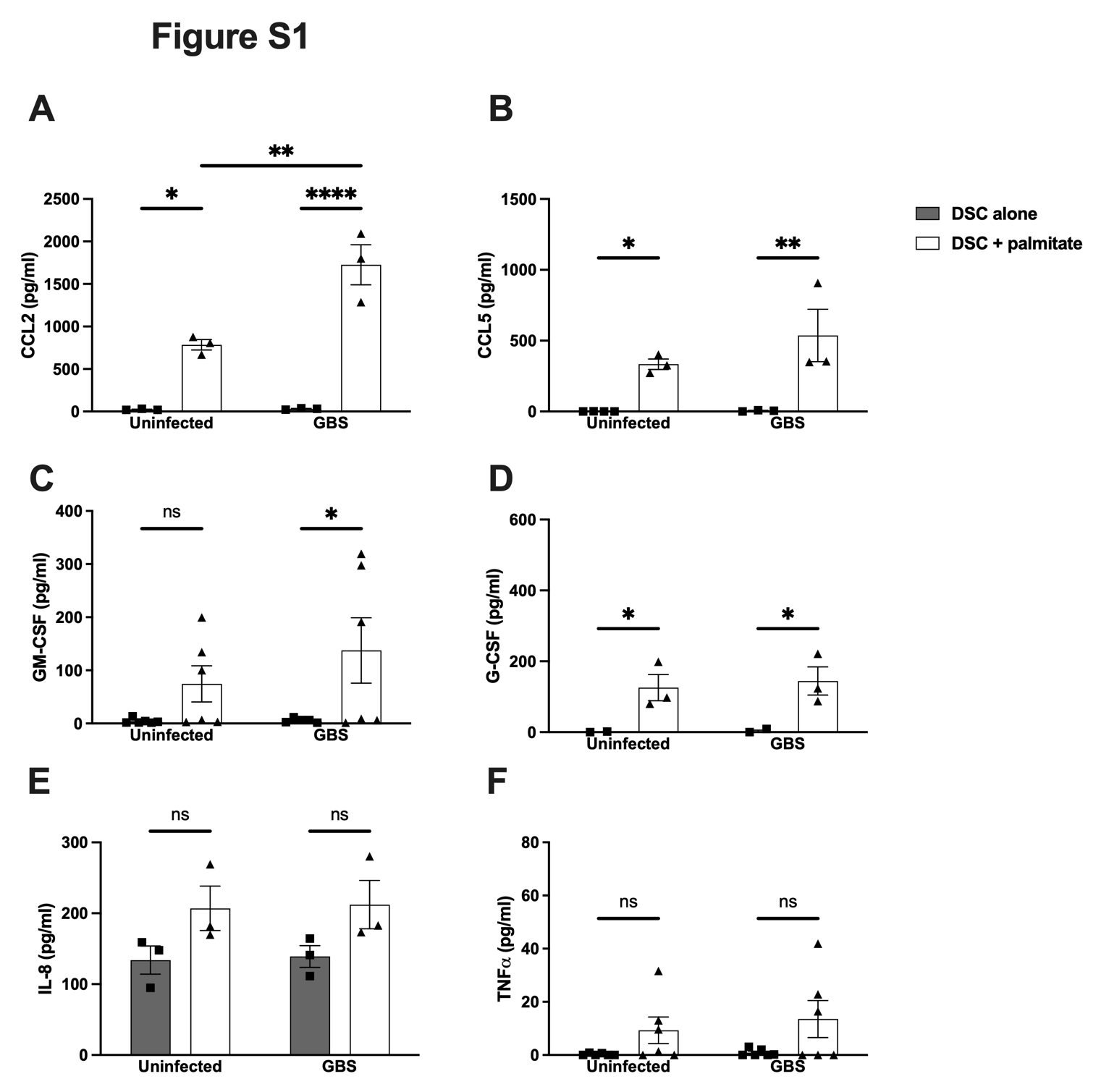

Supplement: Supplementary Figure 1 — Cytokine induction by GBS, palmitate, or both in DSCs infected with GBS and/or treated with palmitate. ELISA analysis of secreted cytokines from DSCs. (A) CCL2, (B), CCL5, (C) GM-CSF, (D) G-CSF, (E) IL-8, and (F) TNFα each followed different patterns of induction by 24 hour infection and/or treatment with GBS and/or palmitate. N = minimum of 3 separate experiments with averaged independent replicates. *p < 0.05, **p < 0.01, ****p < 0.0001 by 2-way ANOVA. ns, not significant. [file Image_1.tiff]

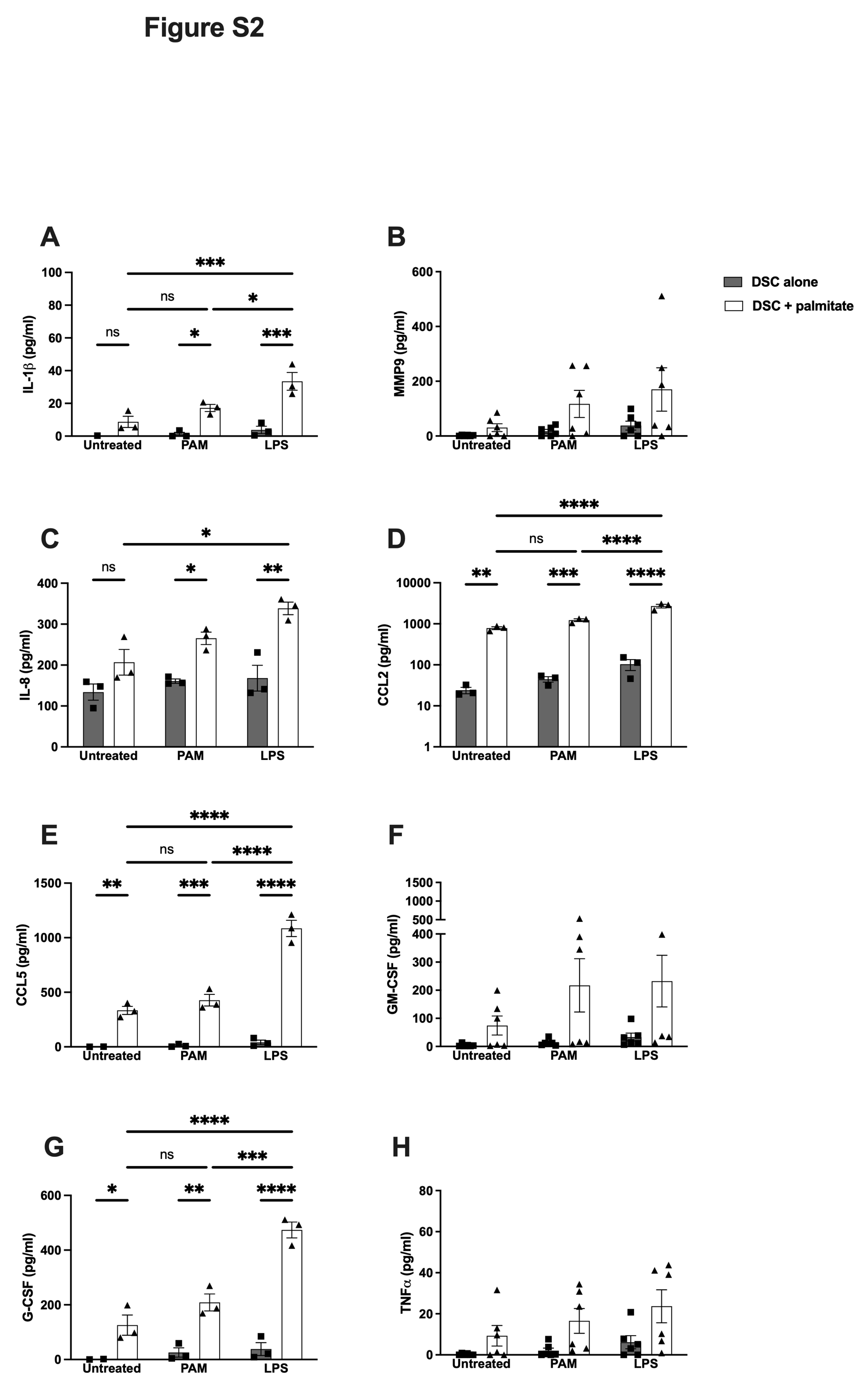

Supplement: Supplementary Figure 2 — Secreted inflammatory protein induction by TLR2 and 4 agonists in conjunction with palmitate in DSCs. (A) IL-1β, (B) MMP9, (C) IL-8, (D) CCL2, (E) CCL5, (F) GM-CSF, (G) G-CSF, (H) TNFα each follow different patterns of induction by 24hr stimulation with TLR2 and 4 ligands PAM3CSK4 (PAM) and LPS, respectively. N = minimum of 3 separate experiments with averaged independent replicates. *p < 0.05, **p < 0.01, ****p < 0.0001 by 2-way ANOVA. ns, not significant. [file Image_2.tiff]

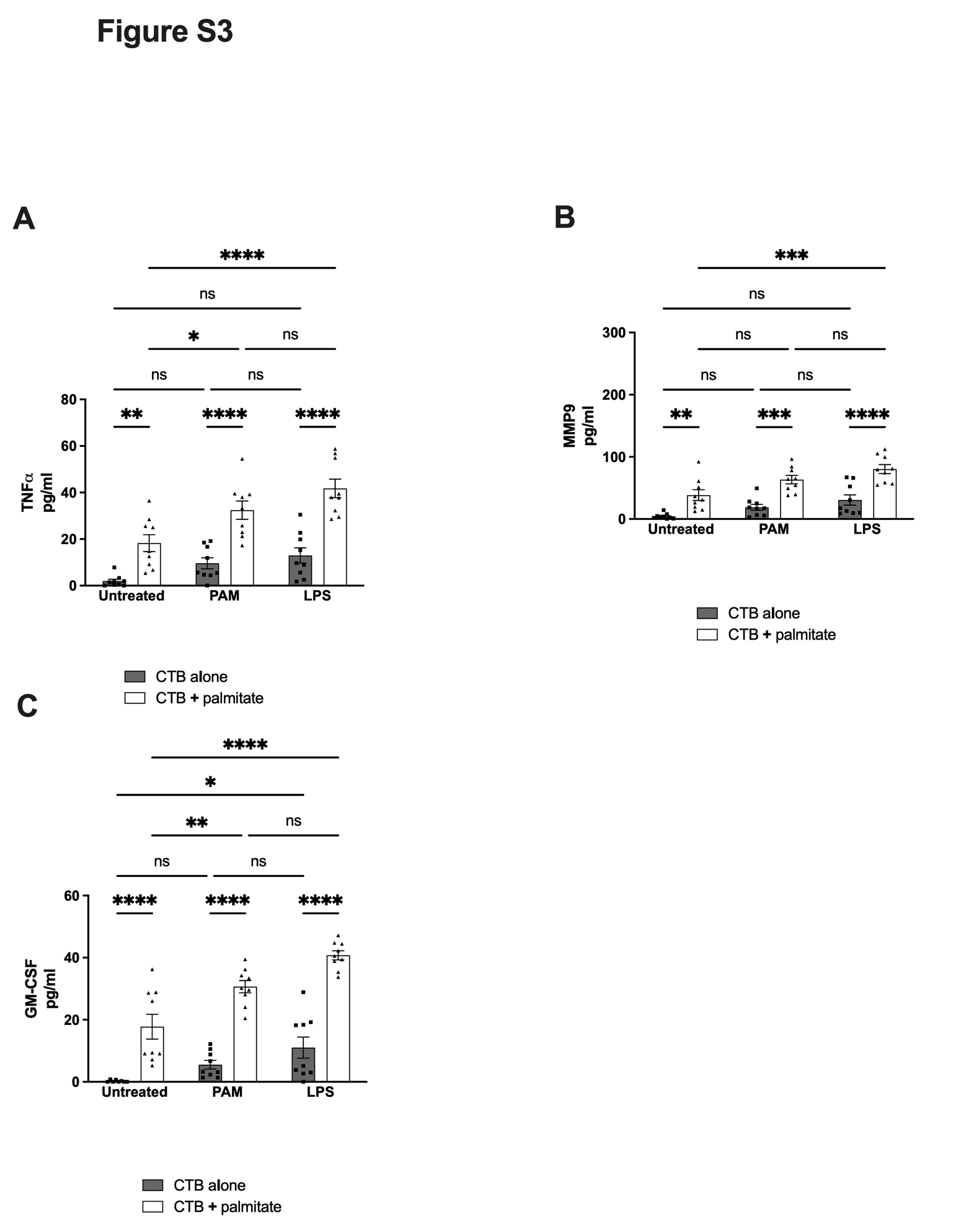

Supplement: Supplementary Figure 3 — Secreted inflammatory protein induction by TLR2 (PAM) and 4 (LPS) agonists in conjunction with palmitate in CTBs. (A) TNFα, (B) MMP9, and (C) GM-CSF. N = minimum of 3 separate experiments with averaged independent replicates. *p < 0.05, **p < 0.01, ****p < 0.0001 by 2-way ANOVA. ns, not significant. [file Image_3.tiff]

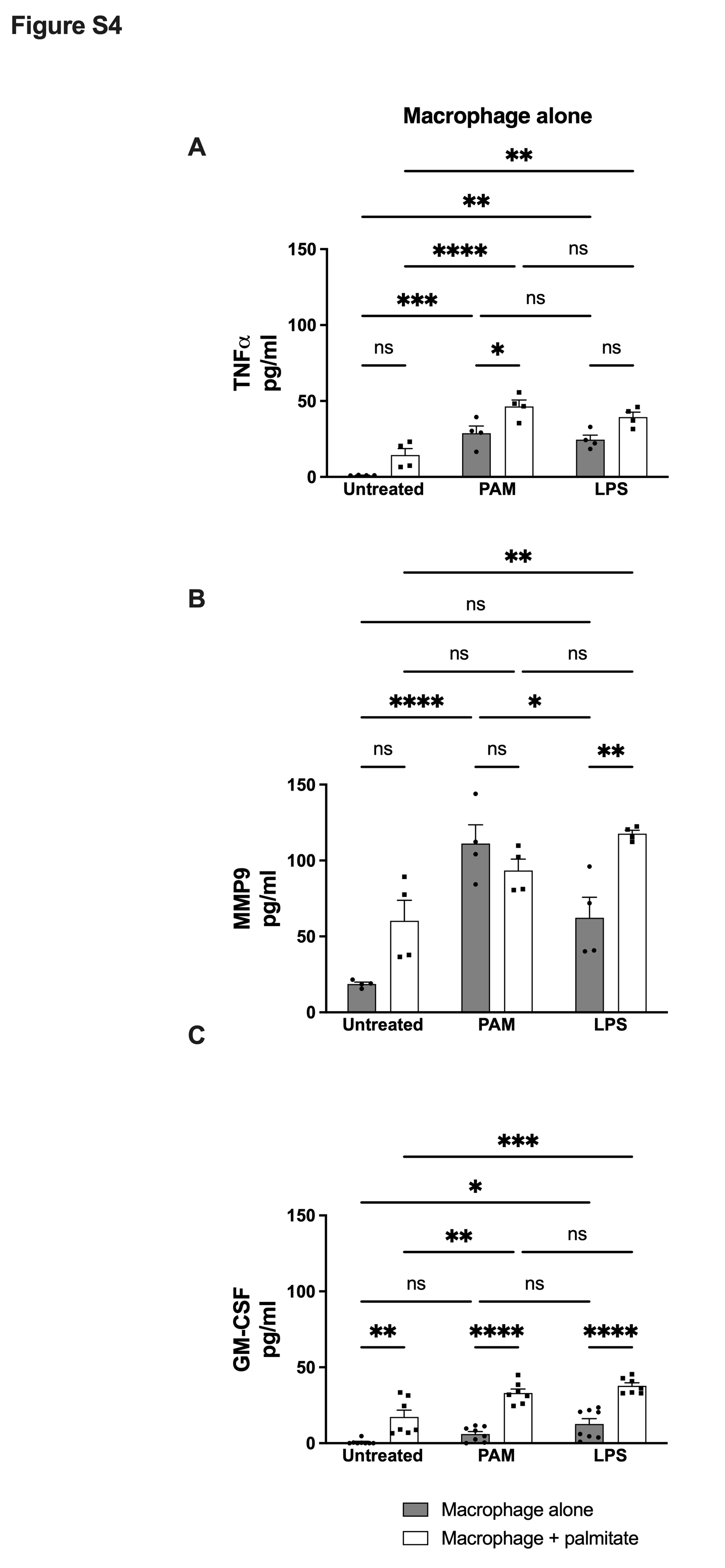

Supplement: Supplementary Figure 4 — Secreted inflammatory protein induction by TLR2 (PAM) and 4 (LPS) agonists in conjunction with palmitate in THP-1 macrophages. (A) TNFα, (B) MMP9, and (C) GM-CSF. N = minimum of 3 separate experiments with averaged independent replicates. *p < 0.05, **p < 0.01, ****p < 0.0001 by 2-way ANOVA. ns, not significant. [file Image_4.tiff]

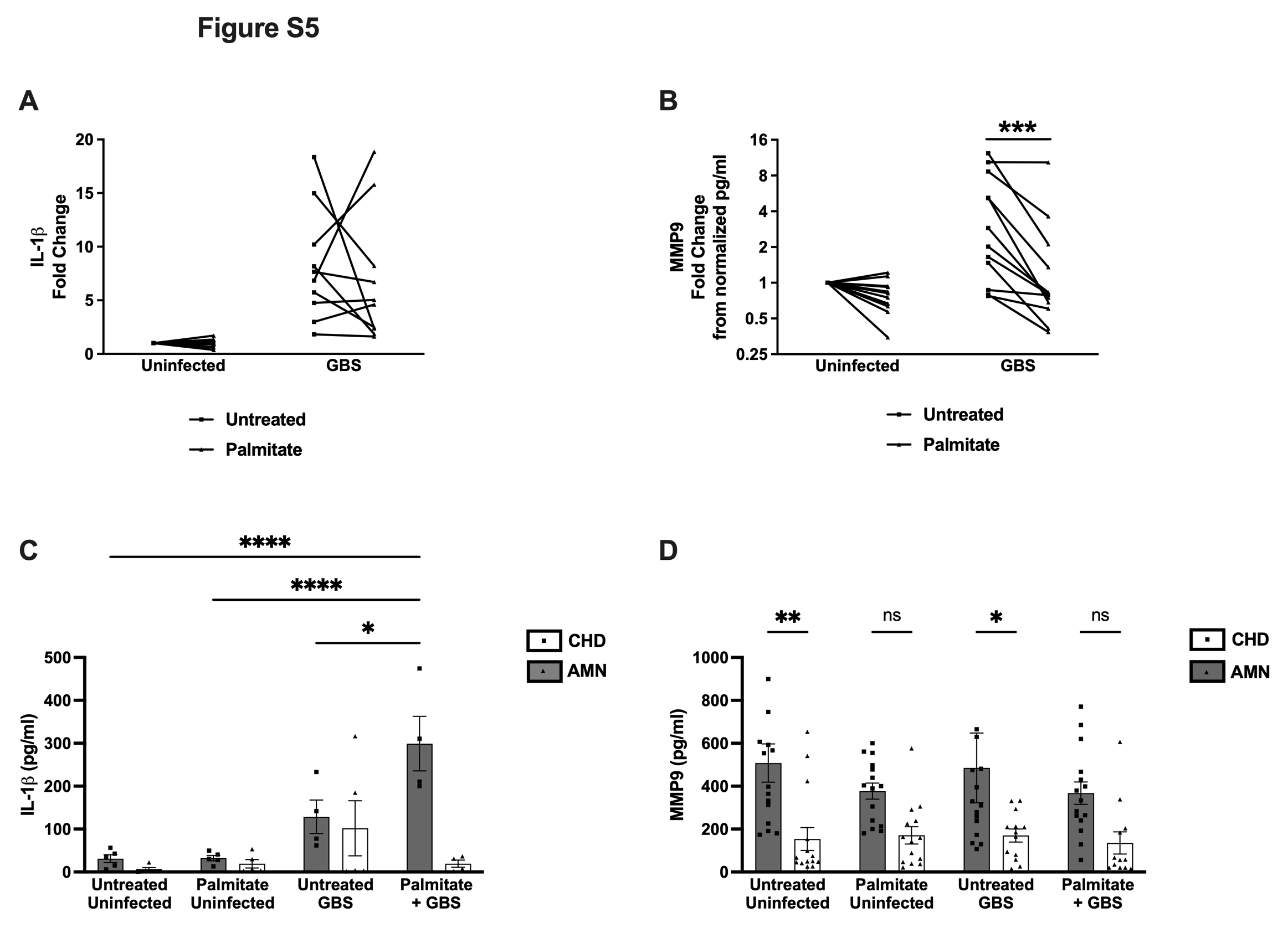

Supplement: Supplementary Figure 5 — Matched sample and side-by-side comparison of IL-1β and MMP9 secretion from membrane punches from human extraplacental membrane.(A) IL-1β and (B) MMP9 ELISAs of 24hr supernatants from individual patient’s membrane punches matched with line between treatments with GBS and palmitate. (C) IL-1β and (D) MMP9 ELISA of CHD and AMN for side-by-side comparison of protein secretion between adjacent extraplacental membrane layers. (A, B) N = 11 independent experiments with averaged independent replicates; (C, D) N = 5 independent experiments with averaged independent replicates. * p < 0.05, **p < 0.01, ***p < 0.001, ****p < 0.0001 by 2-way ANOVA. ns, not significant. [file Image_5.tiff]
