# Supplementary material for: Palmitate and group B Streptococcus synergistically and differentially induce IL-1β from human gestational membranes
Source: Front Immunol. 2024 May 23;15:1409378. doi: 10.3389/fimmu.2024.1409378 (PMC11158625; doi:10.3389/fimmu.2024.1409378)
Supplement: Supplementary file 6 [file DataSheet_1.pdf]

Table S1

Age of mother (years)	race/ethicity of mother	weeks gestation	sex of fetus	BMI of mother	GBS status of mother
35	Caucasian	39	Male	27.6	Negative
28	Hispanic	38	Female	47.28	Positive
29	Black	39	Male	48.82	Negative
23	Black	39	Female	36	Negative
31	Hispanic	39	Male	33.01	Negative
29	Caucasian	39	Male	27.19	Negative
38	Caucasian	38	Female	31.94	Negative
30	Other	39	Female	34.57	Negative
34	Caucasian	39	Male	37.26	Negative
32	Asian	38	Male	34.93	Positive
39	Hispanic	39	Female	30.9	Negative
